# Asparagine Endopeptidase Inhibition Attenuates Tissue Plasminogen Activator‐Induced Brain Hemorrhagic Transformation After Ischemic Stroke

**DOI:** 10.1111/cns.70345

**Published:** 2025-03-21

**Authors:** Guanfeng Xie, Gege Jiang, Liqin Huang, Shangqi Sun, Xiaoyi Li, Bingjie Wu, Hualong Wang, Zhentao Zhang, Keqiang Ye, Ying Yu, Jing Xiong

**Affiliations:** ^1^ Department of Neurology Renmin Hospital of Wuhan University Wuhan Hubei Province China; ^2^ Department of Neurology, Union Hospital, Tongji Medical College Huazhong University of Science and Technology Wuhan Hubei Province China; ^3^ Department of Neurology The First Hospital of Hebei Medical University, Brain Aging and Cognitive Neuroscience Laboratory of Hebei Province Shijiazhuang Hebei China; ^4^ Faculty of Life and Health Sciences, Shenzhen Institutes of Advanced Technology Chinese Academy of Sciences Shenzhen Guangdong Province China; ^5^ Taikang Center for Life and Medical Sciences Wuhan University Wuhan China

**Keywords:** asparagine endopeptidase, blood–brain barrier, hemorrhagic transformation, ischemic stroke, tissue plasminogen activator

## Abstract

**Background:**

Thrombolytic treatment with tissue plasminogen activator (tPA) is one of the approved pharmacological therapies for acute ischemic stroke. However, the use of tPA is limited due to hemorrhagic transformation (HT) and the narrow therapeutic time window. Previous studies demonstrated that asparagine endopeptidase (AEP), a widely expressed pH‐dependent endo‐lysosomal cysteine protease, can induce neuronal death during ischemia‐reperfusion injury. But whether AEP is engaged in HT during ischemia‐reperfusion injury is unclear. In the current study, we expanded the role of AEP on HT after delayed tPA administration.

**Methods:**

In order to investigate the effects of AEP on HT after delayed tPA administration following ischemic stroke, the middle cerebral artery occlusion/reperfusion (MCAO/R) was performed in wild‐type (WT) and AEP knockout (KO) transgenic mice, followed by delayed administration of tPA (10 mg/kg, 3 h after occlusion). Additionally, we explored the potential of R13, a specific TrkB agonist with a strong inhibitory impact on AEP, to mitigate injury induced by tPA. 24 h after tPA administration, the following parameters were assessed: infarct volume, behavioral tests, hemorrhagic levels, Evans blue leakage, tight and adherens junction protein expression, blood–brain barrier (BBB) function, cerebral vascular structure, matrix metalloproteinases (MMPs), and BBB‐regulated protein low‐density lipoprotein receptor‐related protein 1 (LRP‐1) expression. To construct an in vitro model to examine the effects of AEP on ischemia‐reperfusion injury after tPA treatment, human umbilical vein endothelial cells (HUVECs) were exposed to 4 h of oxygen–glucose deprivation (OGD), followed by treatment with tPA (500 ng/mL). 7,8‐dihydroxyflavone (7,8‐DHF), a natural TrkB agonist with an inhibitory effect on AEP, was applied before OGD.

**Results:**

Compared with tPA‐treated WT mice, AEP KO mice treated with tPA showed improved infarct volume, neurological function, brain edema, brain hemoglobin levels, Evans blue leakage, vascular tight junctions, and basement membrane structure combined with reduced AEP expression and activity within the peri‐infarct area. In addition, the mice treated with R13 exhibited protective effects on the BBB. Furthermore, we found that the expression of MMP2, MMP9, and LRP‐1 in the brain was inhibited by both AEP knockout and R13 treatment. Moreover, HUVECs treated with 7,8‐DHF showed improvements in tight and adherens junction proteins and suppressed levels of MMP2, MMP9, and LRP‐1.

**Conclusion:**

Our findings demonstrate that AEP exacerbates HT induced by delayed tPA treatment in acute ischemic stroke by activating LRP‐1, MMP2, and MMP9, which disrupts BBB integrity. We further confirmed R13 as a preventive therapy to attenuate HT induced by delayed tPA treatment in acute ischemic stroke. The present study suggests AEP inhibition may serve as a promising strategy to enhance the safety of delayed tPA thrombolysis for ischemic stroke.

## Graphical abstract

R13 blocks the activation of AEP to prevent delayed tPA‐induced hemorrhagic transformation after ischemic stroke.

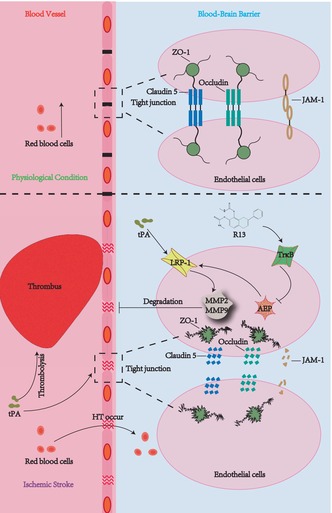

## 1 Introduction

The administration of recombinant tissue plasminogen activator (tPA) within 4.5 h of the onset of acute ischemic stroke is the standard thrombolytic intervention approved by the Food and Drug Administration (FDA) for the treatment of acute ischemic stroke [1, 2]. This intervention is also classified as a class‐I recommendation in the American Heart Association/American Stroke Association (AHA/ASA) guidelines [3]. Unfortunately, the tPA's clinical utility is limited by its narrow thrombolytic time window of 4.5 h [4], even extended to 9 h for selecting patients [5]. A delayed tPA treatment increases the risk of hemorrhagic transformation (HT) and leads to poor clinical outcomes [6, 7, 8]. Thus, it is imperative to develop a therapeutic approach that can extend the therapeutic time window of tPA and reduce the incidence of HT. This advancement is crucial for increasing thrombolysis safety and achieving improved clinical outcomes.

Destruction of the blood–brain barrier (BBB) is a major cause of HT [9, 10]. The integrated structure and normal function of the BBB are maintained by the interaction of cerebral endothelial cells with pericytes, astrocytes, and the basement membrane [11]. This interaction, which is crucial for maintaining the integrity of the BBB, depends on the tight junction proteins (TJPs), including claudins, occluding, junctional adhesion molecule (JAM), and zonula occludens‐1 (ZO‐1) [12]. Degradation of TJPs occurs early in stroke [13], and delayed tPA administration further disrupts the tight junctions and degrades the basement membrane due to the activation of plasmin and matrix metalloproteinases (MMPs) [4, 9]. Therefore, therapeutic strategies that reverse the activation of MMPs are crucial for reducing HT and improving the safety of tPA treatment.

Recent studies have identified a plant‐derived small molecule, 7,8‐dihydroxyflavone (7,8‐DHF), which acts as an agonist for the transmembrane receptor tyrosine kinase B (TrkB) and mimics the role of BDNF. This compound has demonstrated significant inhibitory effects on matrix metalloproteinases (MMPs), specifically MMP1 [14, 15], MMP9 [16], MMP3, and MMP13 [15]. In alignment with the inhibitory effects of MMPs, one of our previous studies demonstrated that 7,8‐DHF and its prodrug R13 [17] also suppressed the C/EBPβ/AEP signaling pathway [18]. Asparagine endopeptidase (AEP), also known as legumain or δ‐secretase, is a cysteine protease that hydrolyzes the c‐terminal substrate of asparagine residues [19]. Activated AEP cleaves nuclear protein inhibiting DNase and leads to neuronal cell death [20]. Our previous studies have indicated that AEP is an important factor contributing to Alzheimer's disease progression [21, 22, 23]. Furthermore, recent studies have established a strong association between AEP and vascular permeability. In oncology, AEP accomplishes tumor migration and invasion by increasing the permeability of vascular endothelial cells [24] and degrading the extracellular matrix [25]. Another research study of thoracic aortic dissection (TAD) found that AEP expressed in TAD tissue from patients and mice was able to induce vascular structure degeneration, dissection, and rupture [26]. AEP also aggravates atherosclerosis pathology by inhibiting the expression of vascular cell adhesion molecule‐1 (VCAM1) in human umbilical vein endothelial cells (HUVECs) [27]. These vascular regulatory functions may be related to the transformation of thrombolytic‐induced HT. Interestingly, AEP was found to have a sharp increase in the peripheral blood from ischemic stroke patients and in the peri‐infarct tissue from rats subjected to middle cerebral artery occlusion (MCAO) [28, 29]. Furthermore, a recent study has found that lysophosphatidic acid exacerbates cerebral ischemia‐reperfusion injury (CIRI) via activating AEP [30]. Apart from the effect on vascular remodeling, AEP also triggers neuroinflammation in several pathological statuses [31, 32, 33, 34]. However, despite sufficient evidence supporting the association between AEP and BBB disruption as well as vascular damage, there is still a lack of evidence to elucidate whether AEP participates in tPA‐induced HT following ischemic stroke.

Here, this study aims to elucidate the role of AEP in the tPA‐induced HT process and determine whether AEP inhibition regulates the occurrence and development of HT. These findings might offer a therapeutic approach to improve the efficacy of thrombolytic therapy.

## 2 Materials and Methods

### 2.1 Animal and Treatment

All experimental procedures were approved by the Institutional Animal Care and Use Committee (IACUC) of Renmin Hospital of Wuhan University, with the IACUC issue number of WDRM animal (welfare) 20240603A. The wile type C57BL/6 mice were obtained from the Animal Experiment Center of Renmin Hospital of Wuhan University. AEP knockout mice were provided by Dr. Keqiang Ye. Male mice aged 8–10 weeks were selected for this research and were housed under specific pathogen‐free conditions in the Animal Experiment Center of Renmin Hospital of Wuhan University. Animal care and handling were performed according to the Declaration of Helsinki and the guidelines of Renmin Hospital, Wuhan University. All the mice (8–10 weeks of ages) except the sham group received MCAO/R surgery (3.5 h occlusion and 24 h reperfusion). 10 mg/kg recombinant human tPA (Actilyse, Boehringer Ingelheim, Germany) or saline was infused via the tail vein at 3 h after occlusion and 0.5 h before reperfusion. Mice used in this part were randomly divided into: WT sham + vehicle group, WT MCAO/*R* + vehicle group, WT MCAO/*R* + tPA group, and AEP KO MCAO/*R* + tPA group (as indicated in Figure 2A). In the part of R13 application, male WT mice received vehicle or R13 (dissolved in 5% DMSO/0.5% methylcellulose) (R13 was kindly gifted from Ye lab [17, 35, 36]) at a dose of 21.8 mg/kg/day, 7 days per week, for 2 weeks by gavage [17]. Mice used in this part were randomly divided into: MCAO/*R* + tPA group, MCAO/*R* + tPA + vehicle (5% DMSO/0.5% methylcellulose) group, MCAO/*R* + tPA + R13 group.

### 2.2 Cell Culture, Oxygen–Glucose Deprivation, and Drug Treatment In Vitro

Human umbilical vein endothelial cells (HUVECs) were obtained from Procell Life Science & Technology (HUVEC‐T1‐CL‐0675). The HUVECs were cultured in complete culture medium for HUVEC (CM‐0122, Procell Life Science & Technology, China). The cells were maintained at 37°C in a humidified atmosphere of 95% air and 5% CO_2_. The OGD model was established as previous studies indicated [37]. In brief, the complete culture medium for HUVEC was removed and replaced with DMEM glucose‐free medium (PM150270, Procell Life Science & Technology). Later, the cells were placed in a modular incubator filled with 5% CO_2_/95% N_2_ for 4 h. Afterward, the DMEM glucose‐free medium was replaced with complete culture medium plus tPA (500 ng/mL) or PBS for another 24 h in a normal incubator filled with 5% CO2 at 37°C [6]. For the 7,8‐dihydroxyflavone (7,8‐DHF) treated group, 0.5 μM 7,8‐DHF was added to the complete culture medium 24 h before OGD [18].

### 2.3 Reagents

The following antibodies and reagents were used: Legumain (AEP) (#93627, Cell Signaling Technology, 1:1000 for western blotting and 1:500 for immunofluorescence), ZO‐1 (YN1410, Immunoway, 1:1000 for western blotting), claudin‐5 (YT0953, Immunoway, 1:1000 for western blotting), occludin (YN2865, Immunoway, 1:1000 for western blotting), JAM‐1 (YT5479, Immunoway, 1:1000 for western blotting), Collagen IV (YM3756, Immunoway, 1:500 for immunofluorescence), CD31 (YM6115, Immunoway, 1:500 for immunofluorescence), LRP‐1 (#64099, Cell Signaling Technology, 1:500 for western blotting), MMP2 (YT2798, Immunoway, 1:1000 for western blotting), MMP3 (YT4465, Immunoway, 1:1000 for western blotting), MMP9 (YT1892, Immunoway, 1:1000 for western blotting), phospho‐TrkB (#4621, Cell Signaling Technology, 1:500 for western blotting), TrkB (#4603, Cell Signaling Technology, 1:500 for western blotting), β‐Actin (#4970, Cell Signaling Technology, 1:2000 for western blotting), anti‐rabbit IgG‐HRP (#70745, Cell Signaling Technology, 1:3000 for western blotting), Alexa Fluor 594‐conjugated goat anti‐mouse IgG (Invitrogen, A‐11005, 1:1000 for immunofluorescence), Alexa Fluor 488‐conjugated goat anti‐rabbit IgG (Invitrogen, A‐32731, 1:1000 for immunofluorescence), DAPI (C1002, Beyotime), TUNEL cell death detection kit (C1090, Beyotime), 2,3,5‐Triphenyl‐2H‐Tetrazolium Chloride (TTC) (G3005, Solarbio), Evans Blue Solution (E8010, Solarbio), Hematoxylin Staining Solution (C0170, Beyotime), Eosin Staining Solution (C0190, Beyotime), Cell Counting Kit‐8 (C0037, Beyotime).

### 2.4 Mouse MCAO/R Model

The experimental model of cerebral ischemia used in this study was the mouse middle cerebral artery occlusion/reperfusion (MCAO/R). This MCAO model was established following the procedure detailed described in our previous work [30]. In brief, mice were anesthetized with 4% chloral hydrate (350 mg/kg, ip), and both the external carotid artery (ECA) and internal carotid artery were surgically exposed. A nylon filament (RWD Life Science, Shenzhen, China) with a thickness of 0.21 mm was inserted through the ECA, advanced 20 mm to occlude the middle cerebral artery from the internal carotid artery, and maintained for 3.5 h before being retracted to trigger reperfusion. Delayed tissue plasminogen activator (tPA, Actilyse, Boehringer Ingelheim, Ingelheimam Rhein, Germany) treatment was administered 3 h post‐occlusion. The rectal temperature of mice was maintained at 36.5°C–37.5°C by a homeothermic heating pad. Mice that showed convulsions, sustained impaired consciousness, or had no apparent contralateral limb dysfunction were excluded from subsequent experiments.

### 2.5 Measurement of Infarct Size

Mice were euthanized and underwent transcardiac perfusion with saline 24 h after reperfusion. The brains were quickly removed and sliced into five serial 2 mm‐thick coronal sections. Brain slices were immersed in 2,3,5‐triphenyltetrazolium chloride (TTC) (2%, G3005, Solarbio) for 20 min. The images were captured with a digital camera, and the infarct volumes were quantified using ImageJ (NIH Image, Bethesda, MD, USA). The infarct percentage was calculated according to a published method [38].

### 2.6 Assessment of Neurological Deficits

Behavioral assessments were conducted by an individual blinded to experimental groups. At 24 h post‐ischemia, results were evaluated using a 5‐point motor function rating scale as outlined earlier [39]: 0 = no neurologic deficit; 1 = paralysis and inability to fully extend the anterior or hind limbs; 2 = circling to the paralyzed side; 3 = falling to the paralyzed side and crawling; and 4 = unable spontaneously to walk and loss of consciousness. The corner test was administered as previously described to assess asymmetries in turning behavior [40]. Performance on the corner test was quantified by the number of left turns out of 10 trials each day.

### 2.7 Assessment of Intracerebral Hemorrhage

A colorimetric assay for hemoglobin (#700540, Cayman Chemical) was employed to measure the hemoglobin levels in brain tissues. Mice were sacrificed under deep anesthesia and infused with 45 mL of cold PBS. The brains were promptly extracted and separated into right and left hemispheres. Hemorrhagic hemispheres were then isolated and washed with ice‐cold PBS solution three times. Subsequently, 1 g of tissue was homogenized with 1 mL PBS solution with 0.16 g/L heparin. Following centrifugation at 10,000 *g* for 10 min, the supernatant was collected for hemoglobin quantification using the provided commercial kit as the manufacturer's guidelines indicated. The absorbance of the hemoglobin standards was recorded using a microplate reader to generate a standard curve. Hemoglobin concentrations in the tissue samples were calculated based on their absorbance values and the standard curve. Finally, average brain hemoglobin levels were determined as the hemoglobin content relative to the protein concentration in the tissue.

### 2.8 Brain Water Content

The wet/dry method was used to determine the water content in the brain, as described in our previous study [41]. Generally, mice were administered an overdose of chloral hydrate, after which the brain was quickly removed and dried in an oven at 110°C for 24 h. The brain water content was calculated using the formula: (wet weight − dry weight)/wet weight × 100%.

### 2.9 Assessment of BBB Integrity

To assess BBB integrity, Evans Blue extravasation was utilized as outlined in previous studies [42]. At 24 h post‐middle cerebral artery occlusion (MCAO), 2% Evans Blue dye (4 mL/kg, Solarbio) was administered through the tail vein. Two hours after the Evans Blue injection, mice were anesthetized with 4% chloral hydrate (350 mg/kg, ip) and subsequently perfused with a 0.01 M PBS solution. The brains were then promptly collected and imaged. The brain tissue was weighed, homogenized in 50% ice‐cold trichloroacetic acid, and centrifuged at 12,000 *g* for 15 min at 4°C to eliminate debris. The resulting supernatants were transferred to fresh tubes. To quantify the Evans Blue in the supernatants, absorbance at 620 nm was measured spectrophotometrically using a microplate reader.

### 2.10 Hematoxylin and Eosin (HE) Staining

Hematoxylin and eosin (H&E) staining was employed to assess morphological changes in the infarcted region. After perfusion with 4% paraformaldehyde (PFA) and paraffin embedding, coronal sections of 10 μm thickness were obtained. The fixed sections were immersed in distilled water for 2 min. Following dewaxing and rehydration, the tissue sections were first stained with hematoxylin solution for 8 min, then briefly exposed to acidic ethanol (1% HCl/70% ethanol) for a few seconds, rinsed with running water to eliminate excess stain, and subsequently stained with eosin solution for 5 min. The sections were then dehydrated using a graded alcohol series before being cleared in xylene. Finally, the slides were mounted with neutral balsam and examined under a microscope. Alterations in the infarct area were observed using a light microscope.

### 2.11 Nissl Staining

For Nissl staining, we utilized the corresponding reagents provided by Servicebio Biotechnology and applied them to paraformaldehyde‐fixed sections in accordance with the manufacturer's instructions. The morphological changes of Nissl bodies and brain hematoma were observed under a light microscope.

### 2.12 AEP Activity Assay

The enzymatic assay for AEP was conducted as previously outlined [18]. In detail, brain and cell lysates were incubated in 200 μL of assay buffer, which consisted of 20 mM citric acid, 60 mM Na_2_HPO_4_, 1 mM EDTA, 0.1% CHAPS, and 1 mM DTT, with a pH of 6.0. The buffer also contained 20 μM of the δ‐secretase substrate Z‐Ala‐Ala‐Asn‐AMC (Bachem). Enzyme activity was assessed by measuring the fluorescence intensity of AMC released upon substrate cleavage. This measurement was performed at an emission wavelength of 460 nm using a fluorescence plate reader, with the reaction carried out at 37°C over a period of 2 h in a kinetic model.

### 2.13 Cell Viability Detection

A Following treatment, HUVECs were maintained in 96‐well plates. The culture medium was refreshed with 100 μL of new complete medium (90 μL of fresh complete medium and 10 μL of CCK‐8 solution added to each well). The plates were then incubated for an additional hour. Absorbance at 450 nm (A450) was measured using a microplate reader. Cell viability was calculated using the formula [37]: cell viability (%) = (experimental group − blank group)/(control group − blank group) × 100%.

### 2.14 Western Blotting Analysis

Mouse brain tissue or cell samples were subjected to lysis in a buffer containing 50 mM Tris, 40 mM NaCl, 1 mM EDTA, 0.5% Triton X‐100, 1.5 mM Na_3_VO_4_, 50 mM NaF, 10 mM sodium pyrophosphate, and 10 mM sodium β‐glycerophosphate, along with a cocktail of protease inhibitors and adjusted to pH 7.4. Following lysis, the samples were centrifuged at 16,000 *g* for 15 min. The resulting supernatant was then mixed with 1 × SDS loading buffer and heated. After separation by SDS‐PAGE, the proteins were transferred onto a nitrocellulose membrane. The membrane was blocked with 5% skim milk for 1 h at room temperature. Subsequently, primary antibodies were applied, and the membrane was incubated overnight at 4°C. After washing the membrane three times with TBST, it was probed with HRP‐conjugated secondary antibodies for 1 h at room temperature. Results were visualized using enhanced chemiluminescent (ECL) substrates.

### 2.15 Immunofluorescence Staining

The brain was sliced into 25‐μm thick sections for the purpose of immunostaining. These sections were first incubated with the primary antibody, followed by incubation with the corresponding Alexa 488‐ or Alexa 594‐conjugated secondary antibody. The primary antibodies were utilized according to the reagent details provided. The stained sections were examined using a fluorescence microscope, and detection and quantification were performed with ImageJ.

### 2.16 TUNEL Staining

The sections were first dewaxed using xylene and then subjected to a graded alcohol series for washing. Following this, they were incubated in a 0.25% Triton X‐100 solution for 20 min. Subsequently, the sections were treated with a TUNEL reaction mixture (G3005, Solarbio) and left to incubate for 1 h in a dark environment. After staining, each section was mounted with a medium that resists fluorescence decay and examined under a fluorescence microscope. The results were expressed as the apoptotic index, calculated by the formula: apoptotic index = (number of positive cells per field/total cells per field) × 100%.

### 2.17 Enzyme‐Linked Immunosorbent Assay (ELISA)

The activity of tPA in the ischemic cerebral hemisphere was detected using the double‐antibody sandwich method. All steps were performed according to the manufacturer's instructions. In brief, brain tissues were collected and lysed. After that, the tissues were centrifuged at 1000 *g* at 4°C for 20 min, then collected 100 μL supernatant was collected. A human Tissue‐type Plasminogen Activator ELISA kit (E‐EL‐H2106, Elabscience) was used for detection. Concentrations were calculated based on a standard curve and expressed in ng/mL.

### 2.18 Statistics

Data analysis was conducted using IBM SPSS Statistics 19 and ImageJ 1.51J8, while GraphPad Prism 5 was employed for visualizing the results. Statistical comparisons were performed using one‐way ANOVA, followed by Tukey's post hoc test for multiple group comparisons when the Levene test indicates homogeneity of variance and Shapiro‐Wilk test indicates normal distribution, or the analysis was performed using the Welch test followed by Dunnett T3 multiple comparisons test when the data do not follow a normal distribution or the variance between groups is different. Statistical significance was considered when the *P* value < 0.05.

## 3 Results

### 3.1 Delayed tPA Treatment Triggers AEP Activation and Aggravates Brain Injury

**FIGURE 1:**
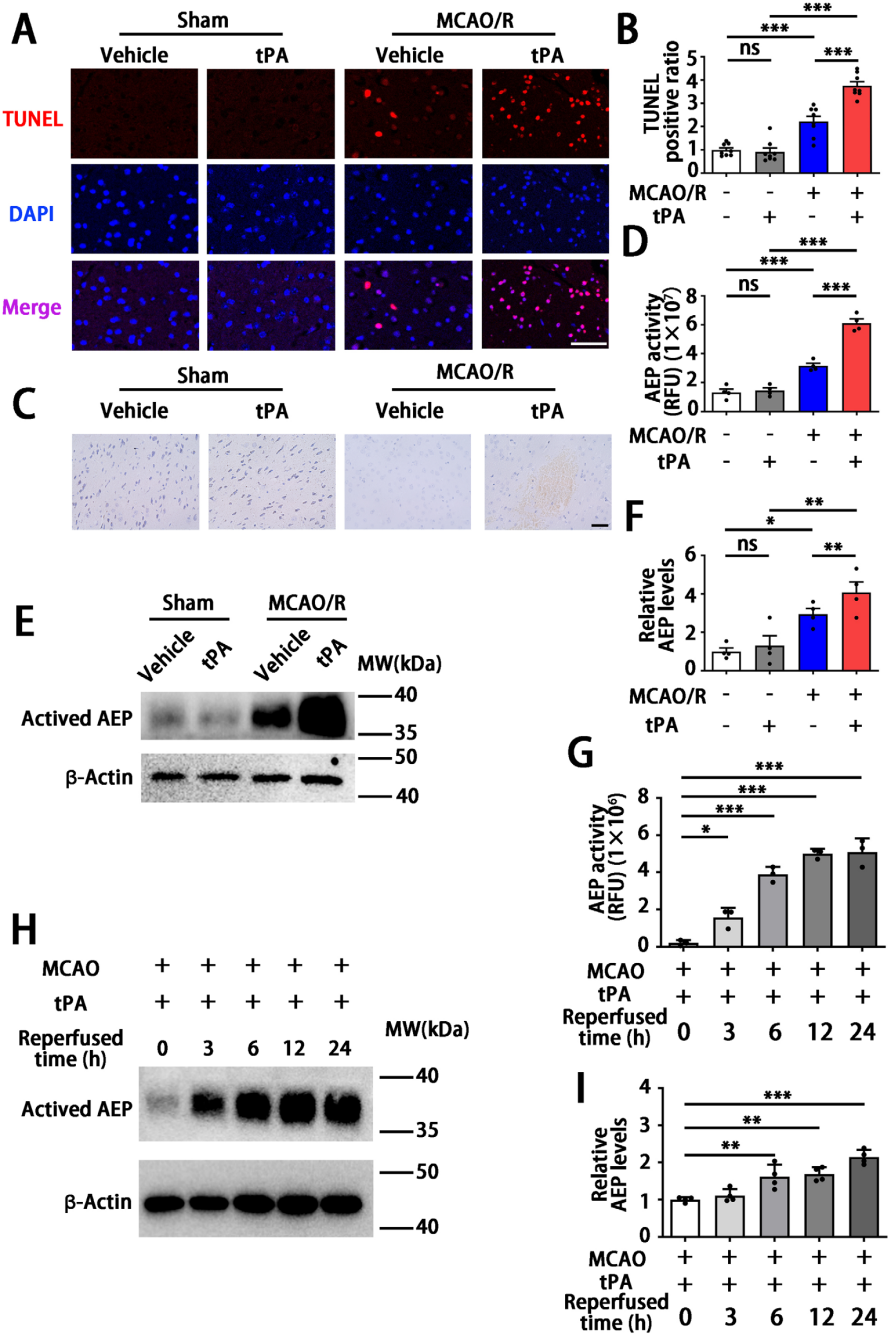
Delayed tPA administration triggers hemorrhagic transformation and activates AEP in the brain. (A, B) Representative TUNEL staining images and relative analysis (scale bar = 100 μm). (C) Representative Nissl staining images (scale bar = 50 μm). (D) AEP enzymatic assay to assess AEP activity in sham or MCAO/R mice with or without tPA treatment (6 h after tPA administration). (E, F) Western blotting to detect the AEP expression in sham or MCAO/R mice with or without tPA (30 mg/kg) treatment (6 h after tPA administration). (G) AEP enzymatic assay to assess AEP activity in MCAO/R followed by tPA injection (30 mg/kg) mice at 0, 3, 6, 12, and 24 h after reperfusion. (H, I) Western blotting to detect the AEP expression in MCAO/R followed by tPA injection (30 mg/kg) mice at 0, 3, 6, 12, and 24 h after reperfusion. Data are presented as mean ± SEM. Statistical analysis is performed using one‐way ANOVA test followed by Tukey's multiple comparisons test. (A), (D), (E), (G), (H) *n* = 4 per group. Normality and variance are assessed via Shapiro‐Wilk test and Levene's test, respectively. *P < 0.05, **P < 0.01, ***P < 0.001.

To investigate whether the alternations of AEP associated with ischemia‐reperfusion injury induced by delayed tPA treatment, we established a widely used rodent model in this study. In this model, tPA is given to mice 3 h after the onset of MCAO, which is equivalent to a 4.5 h delay in clinical settings. This mimics the circumstances of clinical thrombolysis and reperfusion. This delayed tPA‐treated model induces brain hemorrhage in the infarct area but fails to reduce brain infarct size [6, 43]. In our study, TUNEL staining of relative peri‐infarct volume showed that delayed tPA treatment‐induced severe brain injury compared with MCAO/R mice without tPA treatment (6 h after reperfusion), as shown in Figure 1A,B. Furthermore, the Nissl bodies in the cortical region were obviously abundant both in the sham + vehicle group and sham + tPA group, while the MCAO/*R* + vehicle group revealed the formation of cellular vacuoles and loss of Nissl bodies (Figure 1C). Meanwhile, the Nissl staining revealed hemorrhagic transformation occurring in the MCAO + tPA group (Figure 1C). The expression and activity of AEP were significantly elevated within the peri‐infarct area after tPA administration (Figure 1D–F). Subsequently, we evaluated the expression and activity of AEP at different time points following tPA treatment. As shown in Figure 1G–I, the expression of AEP was significantly increased at 6 h after tPA treatment and lasted over 24 h. Besides, AEP activity increased at 3, 6, 12, and 24 h after tPA administration (Figure 1G). Collectively, these data show that delayed tPA treatment increases AEP expression and activity in the ischemia‐reperfusion mouse model.

### 3.2 AEP KO Reduces Delayed tPA‐Induced Brain Impairment After Ischemic Stroke in Mice

**FIGURE 2:**
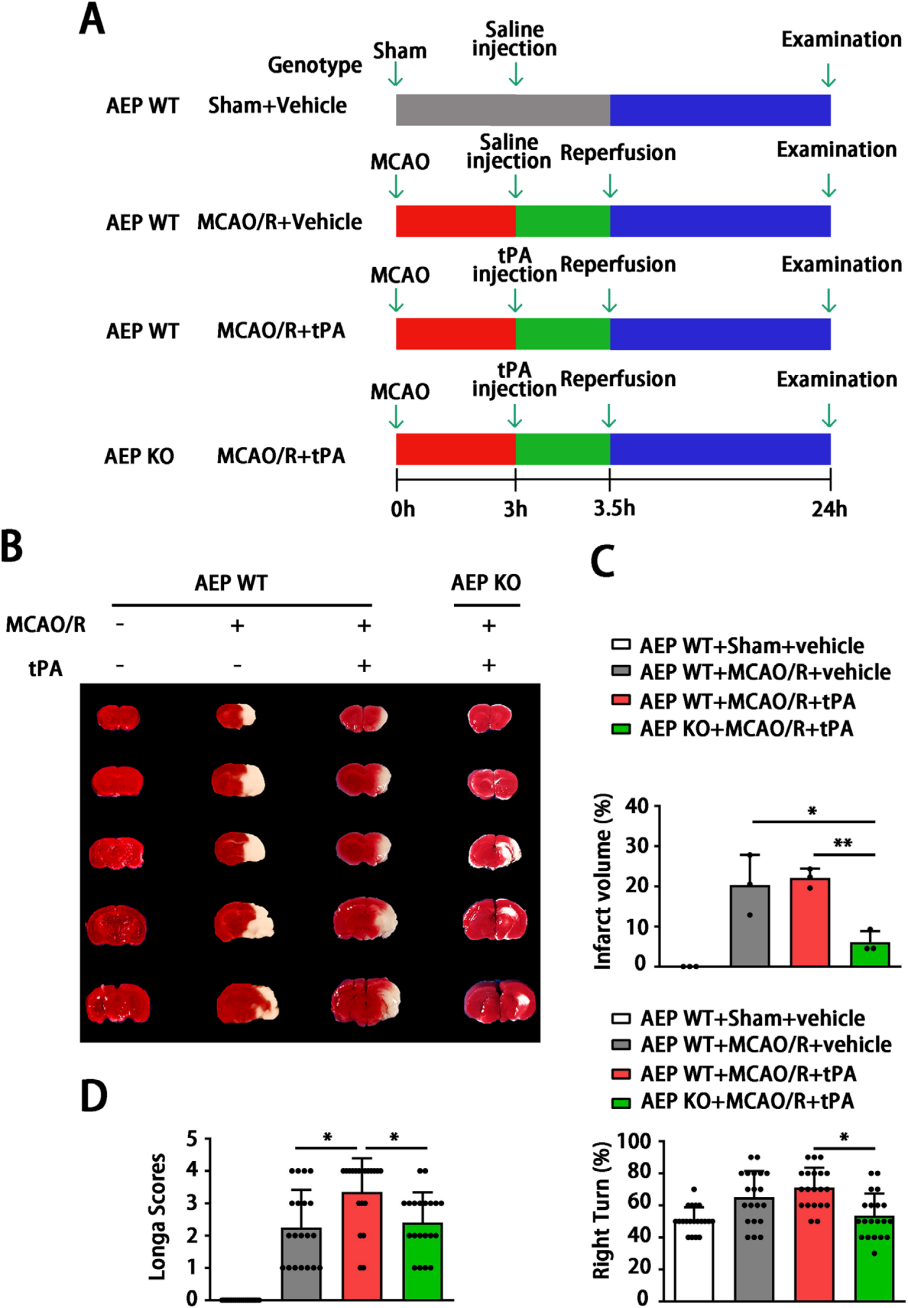
AEP KO ameliorates neurological dysfunction and infarct volume after cerebral ischemia and tPA treatment. (A) Scheme for experimental design in MCAO/R with tPA (10 mg/kg) continuously infused into the vein tail at 3 h after MCAO/R. The timeline for outcome measurements is illustrated. (B, C) Representative images of TTC staining and the corresponding proportion of infarct area. Data are presented as mean ± SEM (one‐way ANOVA test followed by Tukey's multiple comparisons test). (D) Longa Scores and Corner Turn tests are used for sensorimotor function examination. Data are presented as mean ± SEM, and statistical analysis is performed using Welch test followed by Dunnett T3 multiple comparisons test were applied since the *p* value of Levene test < 0.05. (B) *n* = 3, (D) *n* = 20 per group. Normality and variance are assessed via Shapiro‐Wilk test and Levene's test, respectively. *P < 0.05, **P < 0.01.

Since AEP is associated with ischemic events and tPA treatment, to further explore the roles of AEP signaling in delayed tPA‐induced HT, we performed delayed tPA injection in both WT and AEP KO mice after MCAO/R. The mice were randomly divided into four groups: WT sham + vehicle group, WT MCAO/*R* + vehicle group, WT MCAO/*R* + tPA group, and AEP KO MCAO/*R* + tPA group. They received sham or MCAO/R surgery with or without tPA treatment (Figure 2A). 24 h after MACO, we found delayed tPA administration failed to reduce infarct size and induced more severe neurological dysfunction versus the MCAO/R without tPA treatment group (Figure 2B–D). Remarkably, as shown by TTC staining, AEP KO mice exhibited smaller infarct volume versus the WT MCAO/*R* + vehicle group and the WT MCAO/R + tPA group (Figure 2B,C). Furthermore, AEP KO mice showed improved sensory and motor function, as indicated by the Modified Longa Score and Corner Test (Figure 2D). Taken together, these results suggest that AEP knockout provides robust protection against brain damage and neurological dysfunction induced by delayed tPA treatment.

### 3.3 AEP KO Inhibits Delayed tPA‐Induced Hemorrhage After Ischemic Stroke

**FIGURE 3:**
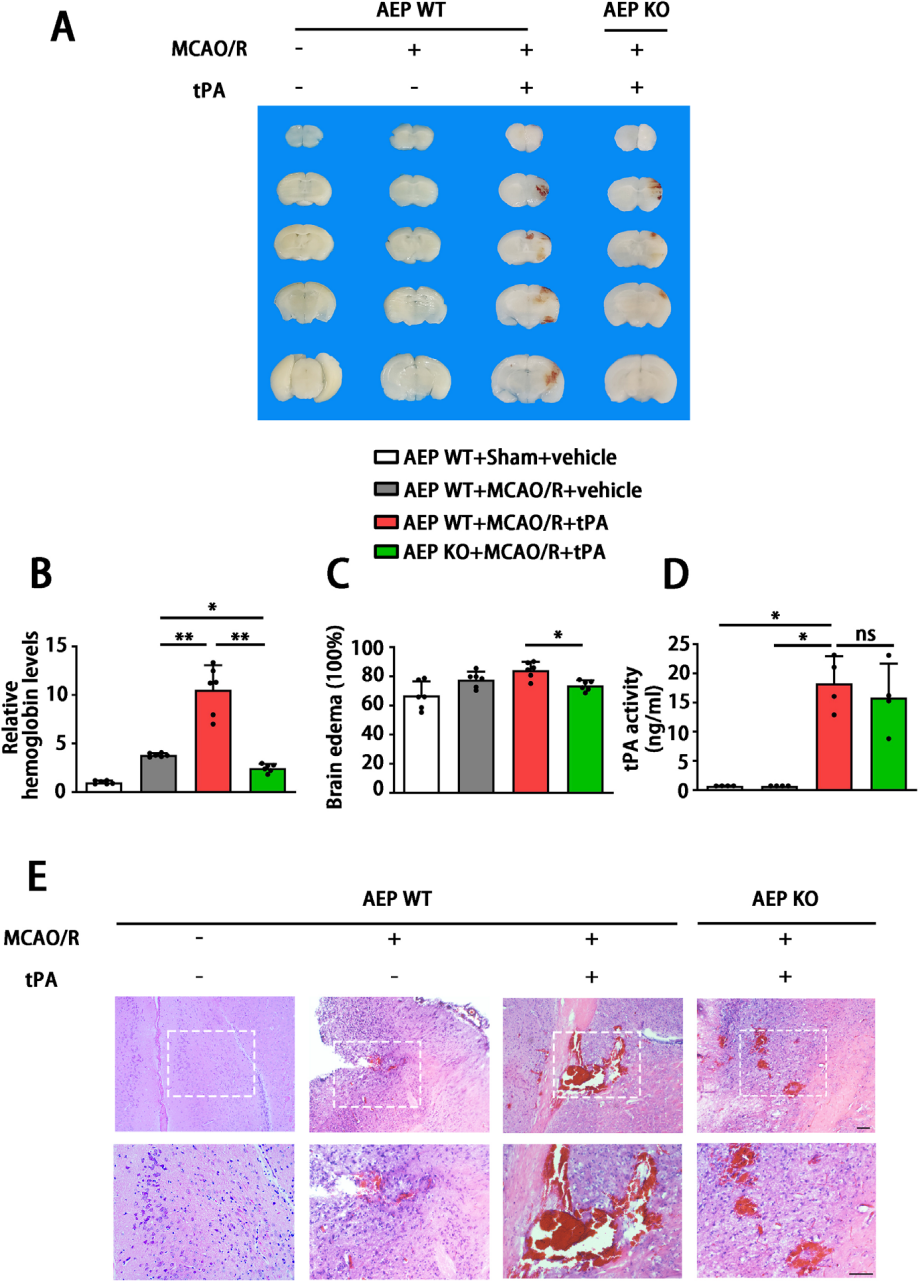
AEP KO ameliorates tPA‐associated hemorrhagic transformation in the stroke mouse model. (A, B) Representative images of hematoma in brain slices and quantification of hemoglobin levels. Data are presented as mean ± SEM and statistical analyses are performed using Welch test followed by Dunnett T3 multiple comparisons test were applied since the *P* value of Levene test < 0.05. (C) Recorded brain water content to assess brain edema at 24 h after delayed tPA administration. Data are presented as mean ± SEM and statistical analysis is performed using one‐way ANOVA test followed by Tukey's multiple comparisons test. (D) ELISA for tPA activity detection. Data are presented as mean ± SEM and statistical analyses are performed using Welch test followed by Dunnett T3 multiple comparisons test were applied since the *P* value of Levene test < 0.05. (E) Representative images of HE staining in the cortical hematoma area (scale bar = 50 μm). (A), (C) *n* = 6, (D) *n* = 4 per group. Normality and variance are assessed via Shapiro‐Wilk test and Levene's test, respectively. *P < 0.05, **P < 0.01.

We subsequently assessed the impact of AEP on tPA‐induced brain hemorrhage in mice. The levels of hemorrhage were assessed. Compared with the WT MCAO/*R* + vehicle group, the WT MCAO/*R* + tPA group exhibited larger hemorrhagic areas, higher hemoglobin levels, and more severe brain edema. In contrast, AEP KO significantly improved these indicators compared to WT mice treated with delayed tPA (Figure 3A–C). Meanwhile, there was no significant difference in tPA activity between WT and AEP KO mice that received delayed tPA administration (Figure 3D). The HE staining showed extensive hematoma, inflammatory cell aggregation, and liquefactive necrosis within the infarct area in the WT MCAO/*R* + tPA group. AEP KO mice demonstrated smaller hematomas and less edema. Interestingly, although the brain slices from WT MCAO/*R* + vehicle mice without tPA treatment did not reveal obvious hemorrhage, the HE representative images showed microhemorrhage in the cortical tissue (Figure 3E). Taken together, these results suggest that the cerebral hemorrhage resulting from delayed tPA treatment can be partly mitigated by AEP KO.

### 3.4 AEP KO Attenuates tPA‐Induced BBB Disruption

**FIGURE 4:**
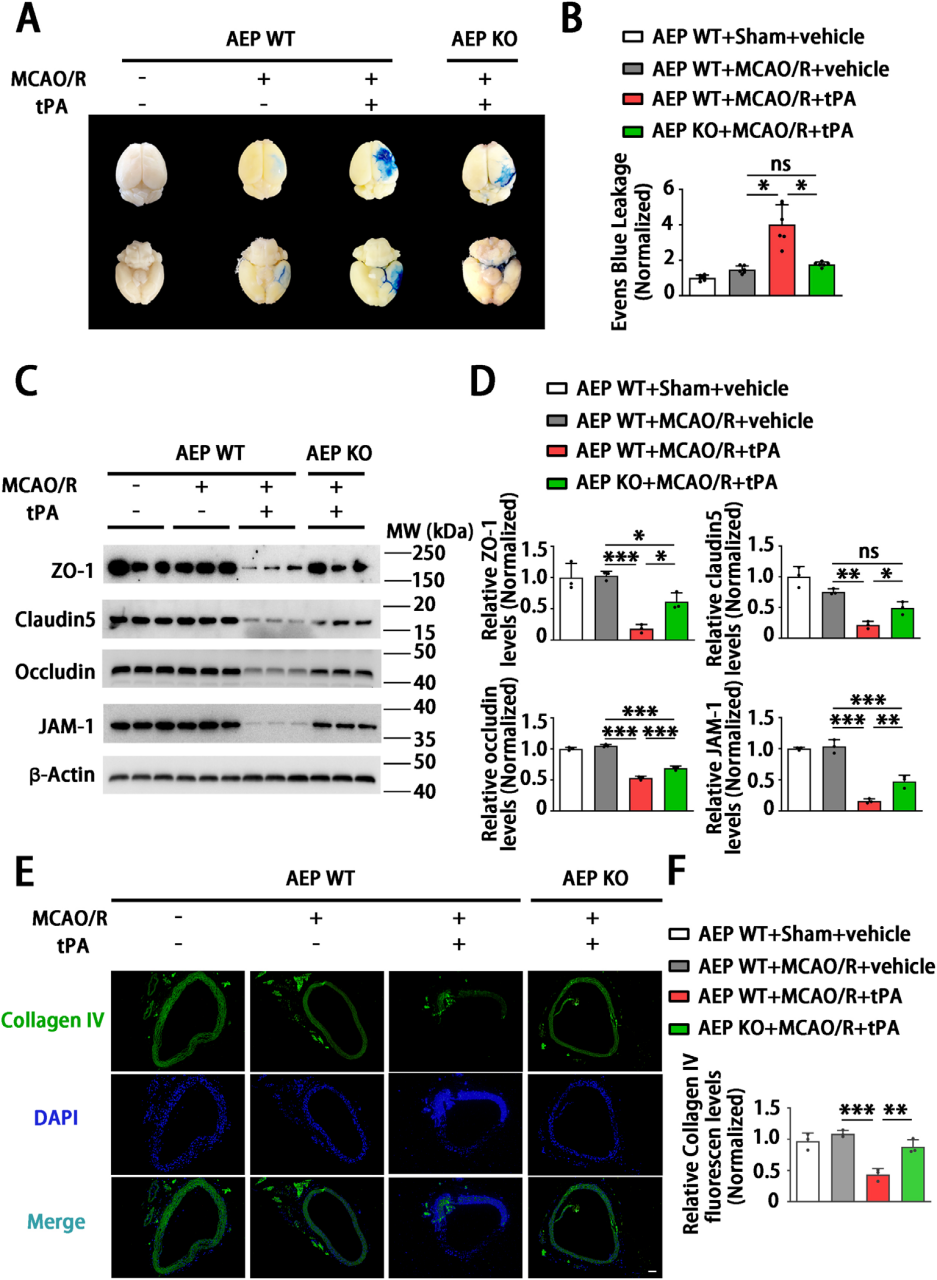
AEP KO attenuates BBB disruption in tPA‐infused stroke mice. (A, B) Representative images and leakage quantification of Evans Blue staining. Data are presented as mean ± SEM, and statistical analysis is performed using Welch test followed by Dunnett T3 multiple comparisons test were applied since the *P* value of Levene test < 0.05. (C, D) Western blotting to evaluate the tight junction proteins ZO‐1, claudin5, occluding, and JAM‐1. Data are presented as mean ± SEM, and statistical analysis is performed using one‐way ANOVA test followed by Tukey's multiple comparisons test. (E, F) Immunofluorescence staining to assess the basement membrane protein collagen IV (Scar bar = 20 μm). Data are presented as mean ± SEM, and statistical analysis is performed using one‐way ANOVA test followed by Tukey's multiple comparisons test. (A) *n* = 6, (C) *n* = 3, (E) *n* = 3 (3 sections and 9 areas) per group. Normality and variance are assessed via Shapiro‐Wilk test and Levene's test, respectively. *P < 0.05, **P < 0.01, ***P < 0.001, ns: Not significant.

To further investigate the role of AEP in delayed tPA‐induced HT, we examined whether AEP influences the integrity and function of the BBB. The WT MCAO/*R* + tPA group displayed greater leakage of Evans Blue dye compared with the MCAO mice that were not treated with tPA (Figure 4A,B). AEP KO mice significantly inhibited the delayed tPA‐induced enhancement of Evans Blue extravasation (Figure 4A,B). Immunoblotting analysis demonstrated that the levels of tight junction proteins (TJPs) (claudin 5, occludin, ZO‐1, and JAM‐1) were downregulated by delayed tPA treatment (Figure 4C,D). AEP KO mice exhibited higher levels of TJPs (Figure 4C,D). Immunofluorescence staining showed that the basement membrane protein collagen IV was decreased and disrupted in delayed tPA‐treated mice. The WT sham + vehicle group, WT MCAO/*R* + vehicle group, and AEP KO MCAO/R + tPA group exhibited elevated collagen IV expression and a complete basement membrane structure (Figure 4E,F). Hence, these data indicate that AEP KO preserves BBB integrity against tPA‐evoked disruptions.

### 3.5 AEP KO Ameliorates Brain Endothelial Damage by Inhibiting LRP‐1, MMP2, and MMP9

**FIGURE 5:**
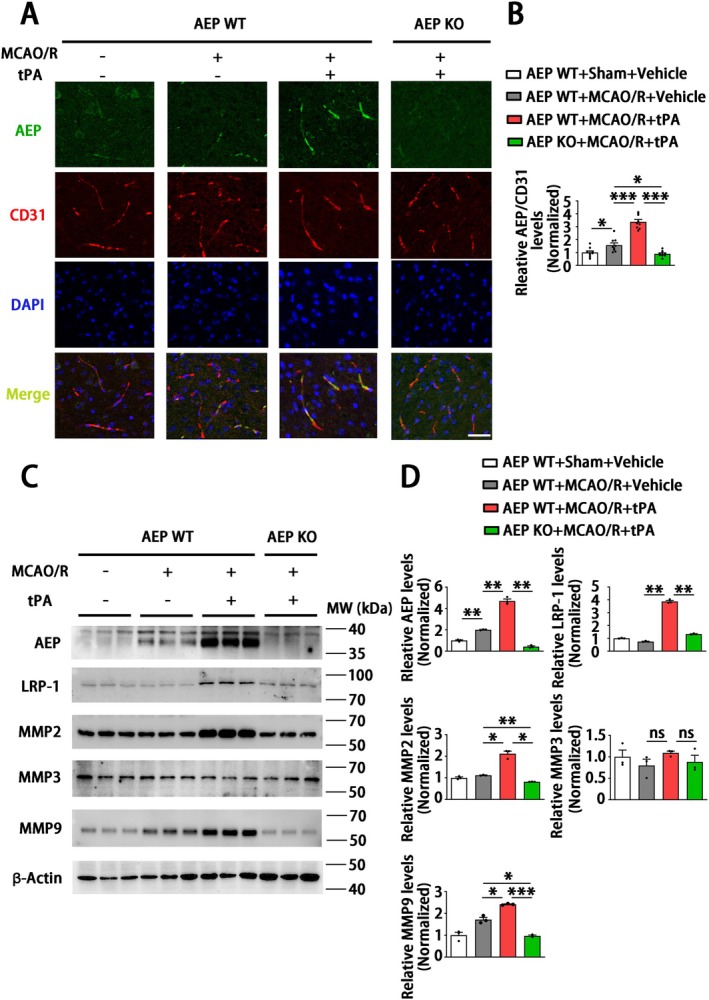
AEP KO suppresses LRP‐1, MMP2, and MMP9 induced by delayed tPA treatment. (A, B) Immunofluorescence staining to assess the expression and location of AEP and CD31 (Scar bar = 100 μm). Data are presented as mean ± SEM, and statistical analysis is performed using a one‐way ANOVA test followed by Tukey's multiple comparisons test. (C, D) Western blotting to evaluate the expression of AEP, LRP‐1, MMP2, MMP3, and MMP9. Data are presented as mean ± SEM, and statistical analysis is performed using the Welch test followed by the Dunnett T3 multiple comparisons test were applied since the *P* value of the Levene test < 0.05. (A) *n* = 3 (3 sections and 9 areas), (C) *n* = 3 per group. Normality and variance are assessed via Shapiro‐Wilk test and Levene's test, respectively. *P < 0.05, **P < 0.01, ***P < 0.001, ns: Not significant.

Endothelial cells are one of the main cell types expressing tight junction proteins (TJPs) and maintaining BBB integrity [44]. Therefore, we further explored the role of AEP on endothelial physiological function after delayed tPA treatment. Immunofluorescence staining showed that AEP significantly co‐localized with endothelial cells in the WT MCAO/*R* + tPA group compared to other groups (Figure 5A,B). Matrix metalloproteinases (MMPs) play essential roles in regulating the connection of TJPs and BBB integrity during tPA‐associated hemorrhage [45]. Therefore, we measured the levels of MMP2, MMP3, and MMP9, which are closely related to tPA‐induced HT. The results showed that AEP expression increased in the WT MCAO/*R* + vehicle group and further elevated in the WT MCAO/*R* + tPA group. Meanwhile, MMP2 and MMP9 also significantly increased in the WT MCAO/*R* + tPA group compared with other groups. However, MMP3 remained unchanged in all groups (Figure 5C,D). Since MMP2 and MMP9 function as important ligands for the low‐density lipoprotein receptor‐related protein 1 (LRP‐1), which is a member of the LDL receptor gene family and acts as a ligand for tPA in the regulation of the BBB [46], we explored its potential involvement in the AEP‐MMPs signaling pathway. Surprisingly, an upregulation of LRP‐1 was observed in the WT MCAO/*R* + tPA group, along with increased levels of MMP2 and MMP9. In contrast, there was a significant downregulation of LRP‐1, MMP2, and MMP9 in the AEP KO MCAO/*R* + tPA group (Figure 5C,D). These results demonstrate that AEP KO may ameliorate delayed tPA‐induced HT and BBB disruption by suppressing the LRP‐1, MMP2, and MMP9 signaling pathway.

### 3.6 7,8‐DHF Ameliorates tPA‐Induced Endothelial Damage in HUVECs Subjected to OGD

**FIGURE 6:**
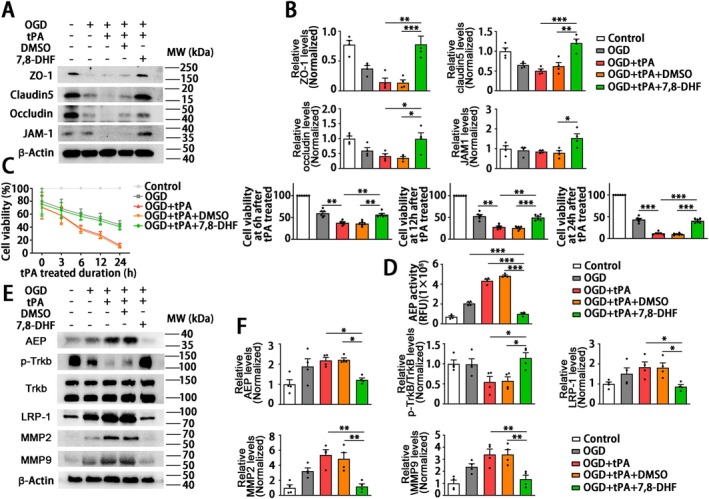
AEP inhibitor 7,8‐DHF protect HUVECs against oxygen–glucose deprivation by inhibiting tPA‐induced elevated LRP‐1, MMP2, and MMP9. HUVECs treated with 0.5 μM 7,8‐DHF for 24 h followed by OGD 4 h and reoxygen‐glucose plus tPA (500 ng/mL) for another 24 h as the methods part described. (A, B) Western blotting to evaluate the expression of ZO‐1, claudin5, occluding, and JAM‐1. (C) The viability of HUVECs at different time points. (D) AEP enzymatic analysis of HUVECs subjected to 24 h tPA treatment following 7,8‐DHF and OGD. (E, F) Western blotting to evaluate the expression of AEP, p‐TrkB, TrkB, LRP‐1, MMP2, and MMP9. (A) *n* = 4, (C) *n* = 6, (D) *n* = 4, (E) *n* = 4 per group. Data are presented as mean ± SEM, and statistical analysis is performed using one‐way ANOVA test followed by Tukey's multiple comparisons test when *P* value of Levene test > 0.05 or Welch test followed by Dunnett T3 multiple comparisons test when the *P* value of Levene test < 0.05. Normality and variance are assessed via Shapiro‐Wilk test and Levene's test, respectively. *P < 0.05, **P < 0.01, ***P < 0.001.

**FIGURE 7:**
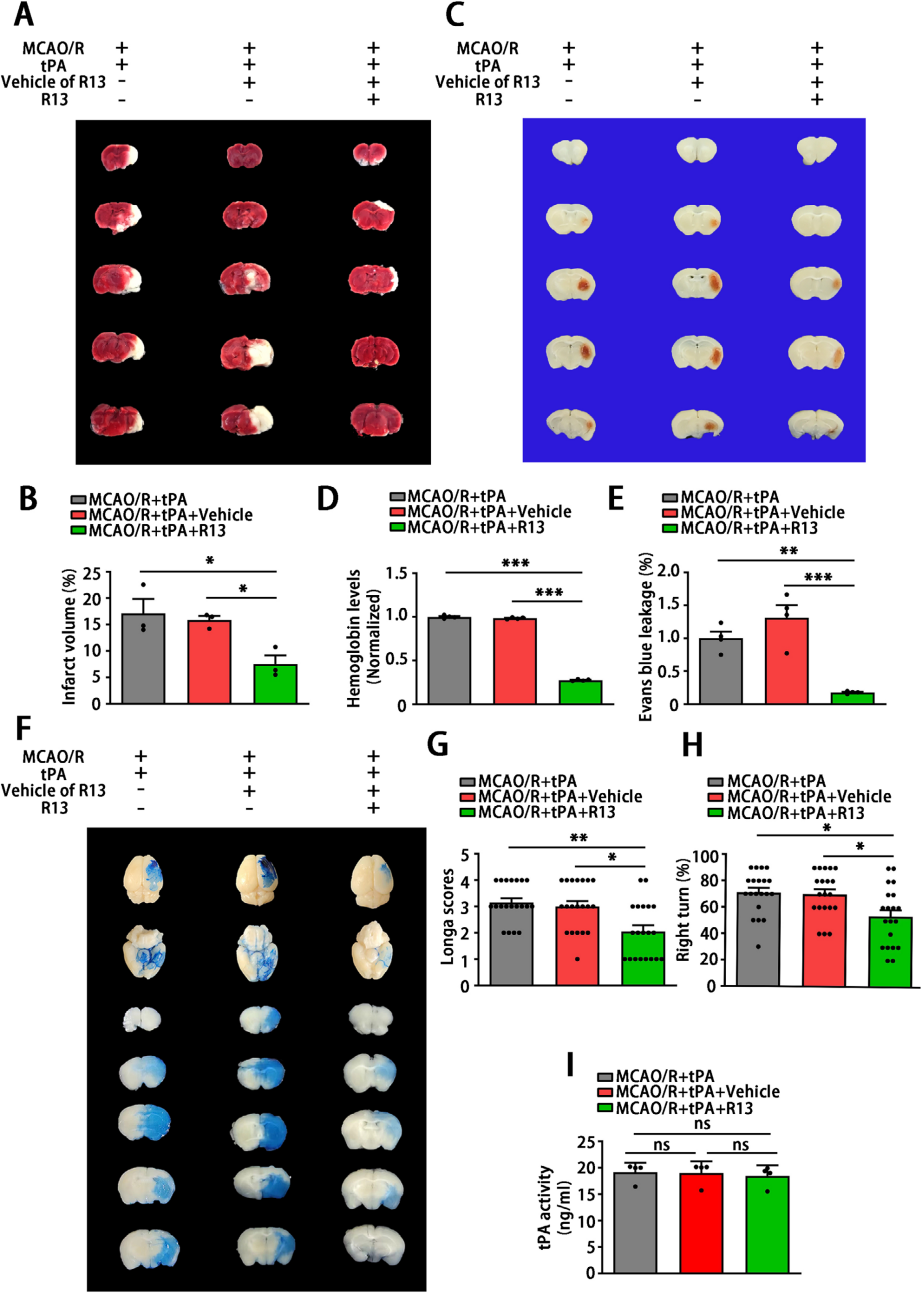
The 7,8‐DHF prodrug R13 confers protection in mice against neurological dysfunction, hemorrhagic transformation, and BBB disruption after cerebral ischemia and tPA treatment. Mice were given R13 (dissolved in 5% DMSO/0.5% methylcellulose) at a dose of 21.8 mg/kg/day, 7 days per week, for 2 weeks by gavage, followed by MCAO/R. Surgery and delayed tPA treatment. (A, B) Representative images of TTC staining and the corresponding proportion of the infarct area. (C, D) Representative images of hematoma in brain slices and quantification of hemoglobin levels. (E, F) Representative images and leakage quantification of Evans Blue staining. (G, H) Longa Scores and Corner Turn tests used for sensorimotor function examination. (I) ELISA for tPA activity detection. (A), (C), (F), (I) *n* = 4, (G, H) *n* = 20 per group. Data are presented as mean ± SEM, and statistical analyses are performed using one‐way ANOVA test followed by Tukey's multiple comparisons test when *P* value of Levene test > 0.05 or Welch test followed by Dunnett T3 multiple comparisons test when the *P* value of Levene test < 0.05. Normality and variance are assessed via Shapiro‐Wilk test and Levene's test, respectively. *P < 0.05, **P < 0.01.

Based on the above results that AEP KO attenuated delayed tPA‐induced BBB disruption by rescuing the endothelial tight junctions, we further explored the mechanism of AEP inhibition on endothelial cell function in vitro. The in vitro BBB disrupted model was established by OGD for 4 h followed by tPA (500 ng/mL) on HUVECs referring to Mao et al. [6]. The AEP inhibition in vitro was achieved by 7,8‐DHF (0.5 μM, 24 h before OGD treatment), a small molecule that activates the BDNF/TrkB signaling and has been proven to inhibit AEP activity in our previous study [18]. The CCK8 revealed that tPA treatment exacerbated the injury of HUVECs subjected to OGD. Remarkably, 7,8‐DHF suppressed the toxicity of tPA and increased the viability of HUVECs at 6 h after tPA treatment (Figure 6C). That is to say, 7,8‐DHF exerts a protective effect on endothelial cells against OGD followed by tPA injury. Endothelial cells conjugated by TJPs form the major barrier of BBB [47, 48]. Therefore, we next verified whether 7,8‐DHF affects the TJPs of HUVECs. As shown in Figure 6A,B, 7,8‐DHF significantly reversed the tPA‐induced inhibitory effect of ZO‐1, occludin, claudin5, and JAM‐1. Furthermore, western blotting also verified the concentration of 7,8‐DHF used in in vitro research was sufficient to activate the TrkB signaling. We also found that tPA induced the activation of AEP, LRP‐1, MMP2, and MMP9 (Figure 6E,F), as same as observed in vivo. Pretreatment of 7,8‐DHF significantly reversed the activation of AEP, LRP‐1, MMP2, and MMP9 induced by tPA (Figure 6D–F). Hence, these in vitro data verify that 7,8‐DHF ameliorates the tPA‐induced endothelial tight junction disruption by inhibiting AEP and suppressing MMP signaling.

### 3.7 7,8‐DHF Prodrug R13 Reduces Ischemic Injury, Preserves BBB Integrity, and Reduces Hemorrhagic Transformation in Mice With MCAO/R Followed by Delayed tPA Treatment

In order to explore the protective effects of AEP inhibition and a potential therapeutic intervention on delayed tPA treatment‐induced HT after stroke, we expanded in vivo studies in mice by administering a 7,8‐DHF prodrug, R13. The R13‐treated mice received R13 at a dose of 21.8 mg/kg/day, 7 days per week, for 2 weeks by gavage before MCAO/R surgery. The TTC staining revealed that oral R13 gavage significantly decreased the infarct volume (Figure 7A,B). To examine whether R13 affects HT, hemoglobin levels were evaluated at 24 h after tPA treatment. The results indicated that R13 treatment attenuated delayed tPA‐induced HT (Figure 7C,D). Furthermore, R13 significantly alleviated the disruption of BBB caused by delayed tPA treatment, as revealed by Evans Blue leakage (Figure 7E,F). Besides, the sensor and motor function of mice that received R13 were rescued, as demonstrated by the Longa score and corner test (Figure 7G,H). These results indicated that R13 ameliorates brain injury and neurological dysfunction within ischemic stroke followed by delayed tPA administration. Additionally, R13 and its vehicle (5% DMSO/0.5% methylcellulose) exerted no effects on the tPA thrombolytic process, since the tPA activity remained unchanged in all three groups (Figure 7I). Taken together, these results suggest that R13 mitigates ischemic injury, preserves BBB integrity, and reduces tPA‐induced brain hemorrhage.

### 3.8 R13 Stabilizes Tight Junctions in the Brain by Suppressing LRP‐1, MMP2, and MMP9 Axis Through AEP Blockade

**FIGURE 8:**
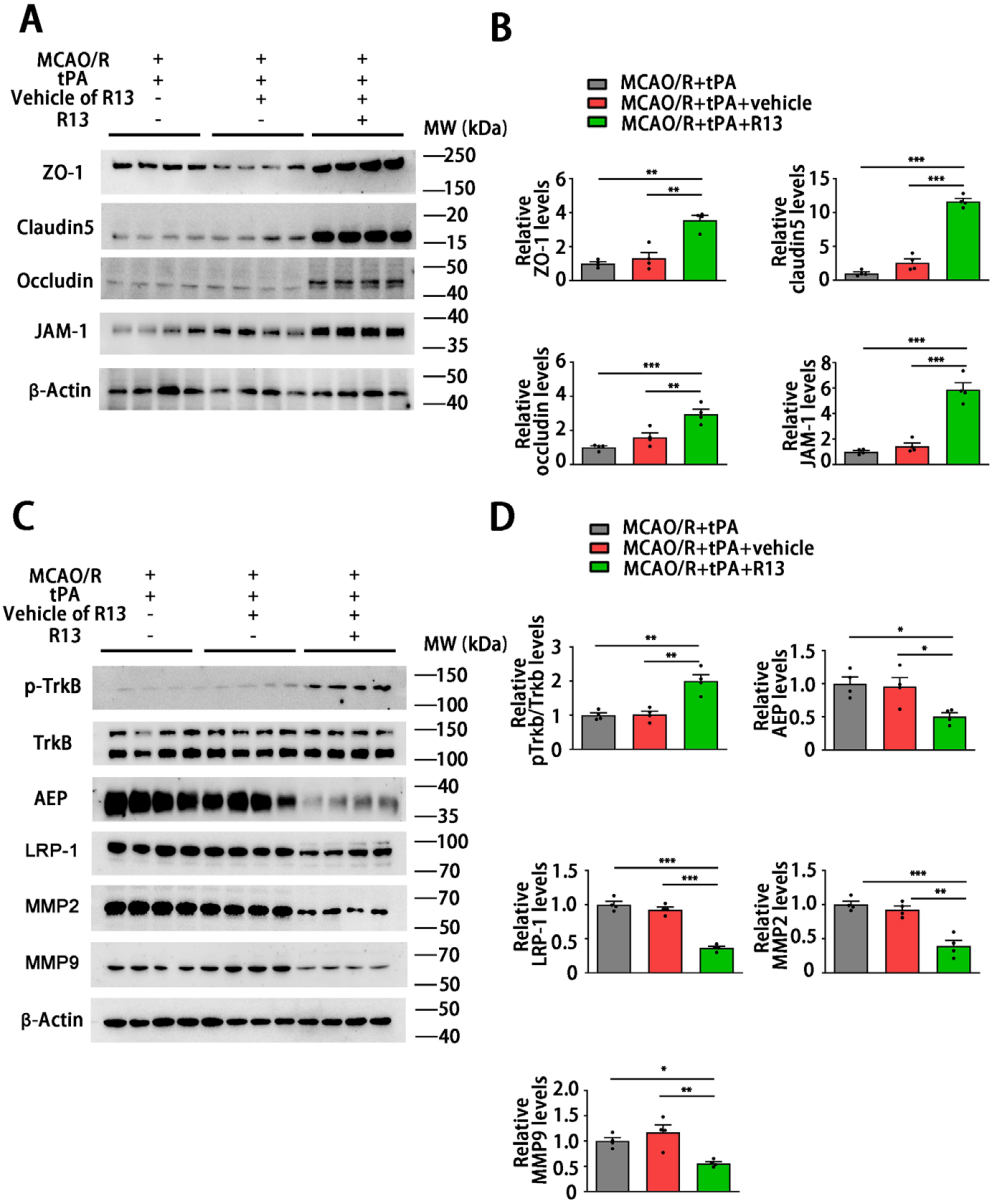
R13 inhibits the activation of LRP‐1, MMP2, and MMP9 induced by delayed tPA treatment to ameliorate the destruction of tight junction proteins destruction. (A, B) Western blotting to evaluate the expression of ZO‐1, claudin5, occluding, JAM‐1. (C, D) Western blotting to evaluate the expression of AEP, p‐TrkB, TrkB, LRP‐1, MMP2, MMP9. (A), (C) *n* = 4 per group. Data are presented as mean ± SEM and statistical analyses are performed using one‐way ANOVA test followed by Tukey's multiple comparisons test when *P* value of Levene test > 0.05 or Welch test followed by Dunnett T3 multiple comparisons test when the *P* value of Levene test < 0.05. Normality and variance are assessed via Shapiro‐Wilk test and Levene's test, respectively. *P < 0.05, **P < 0.05, ***P < 0.001.

To investigate the potential of a preventive strategy for tPA thrombolytic treatment, we subsequently explored the mechanisms by which R13 mitigates the neuronal injury induced by the delayed tPA. The western blotting revealed that the tight junction proteins ZO‐1, Claudin5, occluding, and JAM‐1 significantly elevated in the MCAO/*R* + tPA + R13 group compared with other groups (Figure 8A,B), which indicates that R13 is able to maintain the stability and structure of the BBB. R13 is a prodrug of 7,8‐DHF with improved oral bioavailability and pharmacokinetic profile. It displays a robust effect in activating TrkB and suppressing AEP [17]. We found that the mice brain after R13 treatment displayed TrkB activation and AEP inhibition. R13 also inhibited the expression of the tPA receptor LRP‐1 and the downstream molecules MMP2 and MMP9 (Figure 8C,D). In summary, R13 alleviates delayed tPA‐induced HT by suppressing MMP2 and MMP9 suppression, with these protective effects primarily mediated by the inhibition of AEP and LRP‐1.

## 4 Discussion

Early vascular recanalization is the most crucial strategy for rescuing the ischemic penumbra and improving the prognosis of individuals suffering from acute ischemic stroke [49]. The guidelines for acute stroke management re‐emphasize that tPA should be listed as the first‐line thrombolytic therapy to facilitate early reperfusion [50, 51]. However, the narrow therapeutic window and severe complications remarkably restrict the clinical application of tPA [52, 53]. In the present study, we investigated the effect of AEP on HT and BBB disruption in acute ischemic mice with delayed tPA treatment. We found that AEP knockout and AEP inhibition significantly improved neurological dysfunction and decreased infarct volume in MCAO/R mice followed by tPA administration. We further confirmed that AEP inhibition ameliorated tPA‐induced HT by preserving the integrity of tight junctions in cerebrovascular endothelial cells and facilitating the restoration of the BBB. These protective effects mainly depend on the inhibition of the tPA receptor LRP‐1 and its downstream effectors, MMP2 and MMP9. The clinical implications of these findings suggest that the use of R13 as a preventive therapy in conjunction with tPA thrombolysis could be advantageous for patients suffering from acute ischemic stroke, who are at high risk of HT or those who have passed the thrombolysis therapeutic time window.

The ischemic condition is likely to result in dysfunction of the cerebrovascular unit and disruption of the BBB [54]. The disruption of blood–brain barrier (BBB) integrity results in heightened vascular permeability and hemodynamic disturbances, which initiate a cascade of reactions when tissue plasminogen activator (tPA) treatment is administered. This sequence ultimately contributes to the development of HT and adverse clinical outcomes [55]. Even though some mechanisms underlying tPA‐induced HT have been elucidated, there is still a lack of a potent strategy to deal with this complication caused by tPA thrombolysis [56]. Thus, a novel strategy to maintain BBB integrity is essential to reduce the risk of intracerebral hemorrhage induced by tPA treatment. In the current research, MCAO/R mice receiving delayed tPA treatment showed more severe BBB dysfunction, brain edema, and higher brain hemoglobin levels (Figures 3 and 4). Although delayed tPA treatment had no effect on infarct volume (Figure 2B,C), it was associated with a deterioration in neurological function (Figure 2D), reminding us that BBB dysfunction and HT limit the thrombolytic efficacy of tPA [10]. The tPA thrombolysis rescues ischemic penumbra by dissolving thrombus and achieving cerebrovascular recanalization. However, besides its intended role in clot lysis, tPA also triggers the activation of several proteases and signaling molecules, thereby disrupting the homeostasis of the central nervous system [9]. For instance, tPA may amplify potentially excitotoxic calcium currents by interacting with the NMDA‐type glutamate receptor [57] and degrade extracellular matrix integrity and increase the risk of neurovascular unit dysfunction by exacerbating MMPs' functions after stroke [58]. Furthermore, direct intraventricular injection of tPA to mice without MCAO surgery also induced BBB disruption in an MMP‐dependent manner [59]. In the present research, a delayed tPA treatment model was established by tPA tail vein injection 3 h after occlusion and 30 min before reperfusion (Figure 2A). This protocol was designed to monitor clinical thrombolysis and reperfusion, with the aim of isolating and examining the side effects of tPA, independent of its therapeutic clot‐lysing effects. The concentration (30 mg/kg) of tPA and delayed administration (3 h after the onset of MCAO in mice; equivalent to 4.5 h in ischemic stroke patients) have been shown to induce hemorrhagic transformation but fail to reduce infarct size in several studies [6, 43, 60, 61, 62, 63]. Our results further corroborated the validity of this model. The delayed tPA administration induced intracranial hemorrhage and BBB dysfunction but failed to improve infarct size and neurological function when compared with MCAO/R mice without tPA treatment (Figure 1A–C and Figures 2, 3, 4).

The HT following cerebral infarction is divided into spontaneous hemorrhage and therapeutic‐related hemorrhage [64]. Spontaneous hemorrhage is more likely to occur 7–14 days after stroke onset, presenting as abnormally permeable capillaries and forming cerebral microbleeds [65]. The tPA‐induced hemorrhage always occurs within 24 h and manifests as microartery rupture, resulting in the formation of cerebral hematoma [66]. Interestingly, a significant aspect of this research is that, although the coronal sections revealed no obvious hematoma in MCAO/R mice without tPA administration, the hemoglobin assay and HE staining revealed microbleeds within the cortical area (Figure 3). Besides, the endothelial tight junction and the basement structure collagen IV of this group remained relatively intact compared with the MCAO/*R* + tPA group (Figure 4C–F). Meanwhile, we noticed that AEP was activated by MCAO/R (Figure 1G–I) and further elevated with delayed tPA administration (Figure 5A,B). The tPA receptor LRP‐1, MMP2, and MMP9 were also activated in the MCAO/*R* + tPA group rather than in the MCAO/R without tPA group (Figure 5C,D). Notably, AEP knockout significantly abolished the activation of LRP‐1 combined with MMP2 and MMP9 and maintained BBB integrity (Figures 4 and 5). In general, AEP, which is activated by ischemia/reperfusion conditions within the central nervous system, may participate in spontaneous cerebral HT via a mechanism independent of LRP‐1 and MMPs (most likely neuroinflammation or oxidative stress [33, 67, 68]). Nevertheless, AEP translocates to cerebral endothelial cells to activate LRP‐1, MMP2, and MMP9 when delayed tPA is administered (Figure 5). The activated MMP2 and MMP9 exacerbate endothelial tight junctions and basement membrane lysis, ultimately leading to lethal cerebral HT.

AEP, also known as asparagine endopeptidase or δ‐secretase, is a lysosomal cysteine protease that is known for its ability to cleave both amyloid precursor protein (APP) and Tau, thus contributing to Alzheimer's pathology [69, 70]. Rong et al. [29] verified that, compared with non‐progressive patients, AEP was elevated in patients with progressive ischemic strokes, indicating that AEP is associated with a poor prognosis in ischemic patients. In the present study, we found that MCAO/R significantly activated AEP (Figure 5C,D), consistent with our previous study that ischemic conditions induced by OGD can stimulate AEP activation to impair neurons [30]. Additionally, AEP KO significantly improved brain infarct size and sensory and motor dysfunction (Figure 2B–D). The elevated AEP induced by MCAO can trigger immune cell invasion [28] and cleave an inhibitor of protein phosphatase 2A (PP2A) to aggravate neuronal and synaptic injury [71]. These findings support the neuroprotective effects of the AEP inhibitor exhibited in our results. Furthermore, our findings indicate that the combination of MCAO/R with delayed tPA administration further triggered AEP activation (Figures 1 and 5). In a manner similar to tPA, AEP regulates multiple extracellular matrices to influence vascular permeability [24, 25, 26, 27]. AEP KO abolished the activation of LRP‐1, MMP2, and MMP9 induced by tPA, thereby ameliorating BBB dysfunction and HT (Figures 3, 4, 5).

It is noteworthy that LRP‐1 plays a complex role in ischemic stroke. LRP‐1 is a core component of the neurovascular unit in the blood–brain barrier under physiological conditions [72]. Several studies suggested that LRP‐1 is one of the targets leading to BBB dysfunction [73, 74] and demyelination [75] after ischemic stroke. However, Zhou et al. [76] recently claimed that astrocytic LRP‐1 facilitates the transfer of mitochondria to neurons and alleviates brain ischemic stroke by inhibiting ADP‐ribosylation factor 1 (ARF1) lactylation. Another study reported that LRP‐1 can be used as a targeted carrier of carnosine polymersomes to alleviate ischemic injury [77]. These findings indicate that LRP‐1 plays a multifaceted role in the pathological process of stroke, and non‐selective inhibition may lead to adverse effects. Contrary to our findings, Liberale et al. [78] hold the view that LRP‐1 blockade is ineffective in improving tPA‐induced brain HT. We noticed that LRP‐1 blockade used in this research is achieved through a broad LDLR antagonist, receptor‐associated protein (RAP). RAP is a small intracellular chaperone that prevents premature ligand binding to LRP‐1 and other LDLRs [79, 80]. As discussed above, RAP is a potent inhibitor of LRP‐1, but strong, non‐selective inhibition of LRP‐1 may have varying effects on stroke and tPA‐induced HT. In the present study, we found that LRP‐1 was downregulated with AEP inhibition (Figures 5 and 8). Furthermore, the LRP‐1 of endothelial cells significantly increased when AEP was upregulated in endothelial cells (Figures 5 and 6). That is to say, tPA treatment concurrently causes elevated expression of LRP‐1 and AEP in the endothelial cells around the infarct area. AEP inhibition will reverse the tPA‐induced upregulation of LRP‐1 in endothelial cells. The LRP‐1 suppression mediated by AEP inhibition may be a suitable and gentle approach to restore the BBB function and structure. Unfortunately, we have not yet determined how AEP affects the expression and function of endothelial LRP‐1, which remains a limitation of our study. AEP and LRP‐1 both influence the regulation of the extracellular matrix, Aβ deposition, and apolipoprotein E (ApoE) synergy [22, 23, 46], which indicates that these two proteins may exist in the crosslink between vascular disease and Alzheimer's disease. Regrettably, currently, there is still lack of research to elucidate the interaction between LRP‐1 and AEP. This kind of research may provide promising perspectives to illuminate the relationship between Alzheimer's disease and cerebrovascular disease.

To explore preventive therapy for delayed clinical tPA thrombolysis, we applied R13 in our research. R13 is a prodrug of 7,8‐DHF with a chemical structure modification, which exhibits improved oral bioavailability, pharmacokinetic profile, and BBB penetration rate [17]. Chronic oral administration of R13 activates TrkB signaling and suppresses the activity and expression of AEP, which has been proven to possess functions that inhibit bone loss and delay the progression of Alzheimer's disease in our previous studies [17, 18, 35]. In the current research, R13 treatment significantly activated TrkB phosphorylation and inhibited AEP expression. Similar to AEP KO, R13 treatment also ameliorated the ischemic injury, tPA‐induced BBB dysfunction, and intracranial hemorrhage (Figure 7). TrkB is a downstream target of MMPs and generates negative feedback suppression to MMPs [81, 82, 83, 84]. As a TrkB agonist, 7,8‐DHF exhibits a robust MMPs inhibitory effect [14, 15, 16]. Similarly, in the current study, R13 significantly inhibited the activation of MMP2 and MMP9 (Figure 8C,D), which prevents the delayed tPA‐induced HT. Additionally, although the prodrug R13 showed improved oral bioavailability and BBB permeability [17], it is not totally suitable for the stroke patient due to the impaired swallowing function [85]. Hence, further research focused on the exploration of AEP inhibitors in intravenous formulation or sublingual administration, characterized by significant BBB permeability and rapid intracranial AEP blockade, is required.

In conclusion, delayed tPA administration caused brain damage with hemorrhage and motor dysfunction following MCAO/R. These therapy‐related hemorrhages were associated with AEP. The AEP knockout mice showed mitigated brain injury and hemorrhagic levels. Based on these results, we further explore R13 as a strategy to diminish the delayed tPA‐induced HT. These findings suggest a novel drug for the prevention of HT during thrombolysis in acute ischemic stroke through suppressing the LRP‐1, MMP2, and MMP9, which may pave the way for the development of preventive treatment with tPA thrombolysis after stroke in the pursuit of extending safety for the patient suffering an acute ischemic stroke.

## Author Contributions

J.X. and Y.Y. participated in the study design. G.X. and G.J. designed and performed the animal surgery and data analysis. L.H. and S.S. performed the biochemical experiments. X.L. and B.W. were responsible for the animal behavior test as blind investigators. K.Y. provided the drug R13 applied in this research. J.X. and G.X. participated in manuscript drafting. H.W. and Z.Z. were responsible for proofreading. All authors read and approved the final manuscript.

## Conflicts of Interest

The authors declare no conflicts of interest.

## Data Availability

Data supporting the findings of this study are available upon a reasonable request to the corresponding author.
